# Ethanolic Extract of *Salvia officinalis* Leaves Affects Viability, Survival, Migration, and the Formation and Growth of 3D Cultures of the Tumourigenic Murine HPV-16+-Related Cancer Cell Line

**DOI:** 10.3390/biomedicines12081804

**Published:** 2024-08-08

**Authors:** Alejandra E. Hernández-Rangel, Ariana Cabrera-Licona, Gustavo A. Hernandez-Fuentes, Oscar F. Beas-Guzmán, Francisco J. Martínez-Martínez, Mario A. Alcalá-Pérez, Daniel A. Montes-Galindo, Iram P. Rodriguez-Sanchez, Margarita L. Martinez-Fierro, Juan C. Casarez-Price, Luis De-Leon-Zaragoza, Idalia Garza-Veloz, Iván Delgado-Enciso

**Affiliations:** 1 School of Medicine, University of Colima, Colima 28040, Mexico; ahernandez157@ucol.mx (A.E.H.-R.); gahfuentes@gmail.com (G.A.H.-F.); oscar.beas.11@gmail.com (O.F.B.-G.); 2 Cancerology State Institute, Colima State Health Services, Colima 28085, Mexico; arianacabrera267@gmail.com (A.C.-L.); marioalcalaperez@uaz.edu.mx (M.A.A.-P.); damontesg@gmail.com (D.A.M.-G.); dr.casarezprice@hotmail.com (J.C.C.-P.); drluisdz@gmail.com (L.D.-L.-Z.); 3 Faculty of Chemical Sciences, University of Colima, Coquimatlan 28400, Mexico; fjmartin@ucol.mx; 4 Molecular Medicine Laboratory, Academic Unit of Human Medicine and Health Sciences, Autonomous University of Zacatecas, Zacatecas 98160, Mexico; margaritamf@uaz.edu.mx (M.L.M.-F.); idaliagv@uaz.edu.mx (I.G.-V.); 5 Molecular and Structural Physiology Laboratory, School of Biological Sciences, Autonomous University of Nuevo Leon, San Nicolas de los Garza 66455, Mexico; iramrodriguez@gmail.com; 6 Robert Stempel College of Public Health and Social Work, Florida International University, Miami, FL 33199, USA

**Keywords:** medicinal plants, pharmacognosy, cervical cancer, *Salvia officinalis*, TC-1 cells, 3D-cell culture, flavonoids

## Abstract

*Salvia officinalis* (SO) is one of the most widely used plants in traditional medicine worldwide. In the present study, the effect of an ethanolic extract of *S. officinalis* leaves on hallmarks of cancer of HPV-16-positive cancer tumorigenic cells, TC-1, was analyzed in vitro. Phytochemical and spectroscopic analysis were performed. Additionally, the extract’s flavonoid content, reducing iron, and antioxidant capacity were determined. In regard to the in vitro tests, the cytotoxic activity and its effect on the replicative capacity and on the cell migration of TC-1 cells were analyzed by viability and clonogenic, survival, and wound healing assays. The effect of a pre-treatment or treatment on 3D culture formation, growth, and reversion capacity was also examined. The results of the phytochemical analysis allowed the detection of tannins, saponins, steroids, and flavonoids. The flavonoids content was found to be 153.40 ± 10.68 µg/mg of extract. Additionally, the extract exhibited an antioxidant capacity and a ferric-reducing capacity of around 40% compared to the ascorbic acid. Thin layer chromatographic (TLC) analysis and spectroscopic tests showed the presence of compounds similar to quercetin and catechin flavonoids in the extract. In the in vitro assays, the SO extract induced in a concentration-dependent way changes in cell morphology, the decrease of cell viability, survival, and migration. At a concentration of 125 µg/mL, the extract inhibited spheroid formation, reduced their growth, and affected their reversion to 2D. Ethanolic extract of *S. officinalis* leaves had inhibitory effects on hallmarks of the cancer line HPV-16+. This suggests that the phytochemicals present in it may be a source of chemotherapeutics against cervical cancer.

## 1. Introduction

Human papillomavirus (HPV) is associated with various types of cancers, including anal, cervical, oropharyngeal, penile, vaginal, and vulvar cancers [1]. Among these, cervical cancer is the most common and ranks as the fourth leading cause of death in women [2]. In 2020, the World Health Organization (WHO) estimated a prevalence of 604,000 new cases of cervical cancer and 342,000 deaths worldwide and estimated an increase to 626,275 cases by 2040 [2]. Cervical cancer, like other HPV-related cancers, is caused by persistent infections with one of the 13 cancer-causing genotypes of the human papillomavirus, along with other risk factors [3]. Specifically, high-risk oncogenic HPV types 16 and 18 are responsible for 90% of cases worldwide [1].

Although effective prophylactic vaccines against these HPV types exist, vaccination rates in the Latin American population are low due to social and economic factors [4]. In addition to surgery, chemotherapy, specifically cisplatin, is a primary form of pharmacological treatment for HPV-related cancers [4,5]. Additionally, radiation and concurrent chemoradiotherapy are among the therapeutic modalities being used in clinics, depending on the stage of the disease [6]. While it is effective, it has several adverse effects, such as neurotoxicity, nephrotoxicity, ototoxicity, nausea and vomiting, menstrual disorders, miscarriages, frequent infections, hair loss, and liver and kidney dysfunction [7,8]. On the other hand, resistance to these treatment methods may compromise the efficacy of the overall treatment [6]. Therefore, research into other safer or concomitant treatment alternatives that increase therapeutic efficacy is fundamental. In this regard, phytochemicals are an important source of chemotherapeutics, and the study of chemical components derived from herbal medicines has received increasing attention, especially in recent years [9,10]. They can act both as inhibitors of tumor growth and metastasis, as well as in the antitumor immune response. Furthermore, in combination with other chemotherapeutics, they can have a synergistic antitumor effect, ameliorate adverse effects, or be used to treat complications from chemotherapies [9,10,11]. To date, vincristine, paclitaxel, taxol, vinblastine, and camptothecin, which are plant-derived, are used in clinical practice [12,13].

*Salvia officinalis* (common sage) is one of the main genera of the family Lamiaceae [14]. It is a fragrant evergreen subshrub endemic to southern Europe and Asia but with a worldwide distribution, particularly in the tropical and temperate zones of Central and South America [14,15]. It is widely used in the food industry as a condiment and in traditional medicine. Previous studies of *S. officinalis* have shown it to have antibacterial, fungistatic, virustatic, astringent, hypoglycemic, antinociceptive, anti-inflammatory, antiplasmodial, antioxidant, and anticancer properties [16,17]. Several studies have shown that its active principles are phenolic compounds such as caffeic acid, carnosic acid, carnosol, kaempferol, oleanolic acid, rosmarinic acid, ursolic acid, and the following essential oils: α-pinene, 1,8-cineole, linalool, limonene, borneol, myrcene, β-caryophyllene, spatulanol, β-caryophyllene oxide, viridiflorol, δ-3-carene, and α-bisabolol [17].

Regarding its anticancer properties, the essential oil has been reported to have an antiproliferative and cytostatic effect on the tumor cell lines LNCaP of prostate cancer, MCF7 of breast cancer, HeLa of cervical cancer, and Caco-2 and HT-29 of colon cancer [18,19], while the aqueous extract of the plant inhibited proliferation in HCT15 and CO115 human colon cancer cells [20]. Methanolic extracts have also shown activity in RAW 264.7 and breast cancer lines MCF7 and MDA-MB-231, and in non-Hodgkin’s B-cell lymphoma and human leukemic monocyte lymphoma cells [21,22]. Likewise, the cytotoxic effect of alcoholic extracts on Hep-2 laryngeal carcinoma, HeLa, A-549 lung adenocarcinoma, HT-29 colorectal adenocarcinoma, and A-375 melanoma cells has been characterized as superior to the effect of aqueous extracts [23]. The ethanolic extract of the leaves has been tested in a tumor model of Ehrlich ascites carcinoma as an adjuvant alongside chemotherapy, in acute myeloid leukemia cells, and hepatocellular carcinoma cells [24,25,26]. However, despite previous studies investigating the effects of the ethanolic extract of *S. officinalis* leaves in various cancer models, there remains a lack of specific research on its influence on the characteristics of cervical cancer, especially concerning the TC-1 cell line [27]. Furthermore, considering the widespread use of *S. officinalis* in Mexico, investigating its potential as an adjunct therapy for cervical cancer could offer promising avenues to enhance treatment outcomes and overcome resistance issues. This gap in the scientific literature underscores the need to explore the therapeutic potential of this extract in the context of cervical cancer, aiming to expand our understanding of its potential benefits and mechanisms of action in this disease.

This study aimed to examine, in vitro, the effect of an ethanolic extract of *S. officinalis* leaves on cancer hallmarks of the tumorigenic cervical cancer line TC-1. The study analyzed its cytotoxic and anti-replicative effects, as well as its impact on cell migration and the formation, growth, and reversion capacity of 3D cultures.

## 2. Materials and Methods

### 2.1. S. officinalis Leaf Extract Preparation

*Salvia officinalis* leaves were collected from the Campos community in the municipality of Manzanillo, Colima (19.02640, −104.31412). Taxonomic identification was conducted by comparing it with an herbarium specimen from the National Herbarium of Mexico (MEXU) with catalog number 1304656. 

The leaves were cleaned and dried at 38 °C. The dried leaves were finely ground into a powder. To obtain the extract, 500 g of the powder was subjected to cold maceration in 2L of 96% ethanol by stirring at room temperature on a rotary shaker (HZ-300) for 24 h. The extracted mixture was filtered using Whatman filter paper No. 2 and the filtrate was concentrated using a rotary evaporator (IKA RV 10, Staufen, Germany) at low pressure at 37 °C. The extract residues obtained were collected and quantified, and the yield was determined (%*w*/*w*). The extract was stored at −10 °C until use in an inert atmosphere and according to the protocol of Hernández-Fuentes [28].

### 2.2. Qualitative Phytochemical Analysis of S. officinalis Leaf Extract

The methods outlined by Oloya et al. in 2021 were employed. In brief, for tannin determination, a saturated solution of ferric chloride (FeCl_3_) and gelatin hydrolysis were utilized; for flavonoids, concentrated HCl (Shinoda) and magnesium and Salkowski tests were performed. Alkaloids were identified using the Dragendorff, Mayer, and Wager tests [29], while the hemolysis test with 7% blood agar and foam in water presence were employed for saponins. Steroid rings were revealed through a reaction with H_2_SO_4_ in a chloroform solution; for coumarins, a reaction with NaOH was employed [30]. All determinations were conducted using a stock of the extract at a concentration of 5 mg/mL. 

### 2.3. Spectroscopic Analysis of S. officinalis Leaf Extract

UV data were obtained using an Evolution 300 spectrophotometer (Thermo Fisher Scientific, Waltham, MA, USA) in a MeOH solution. FTIR spectroscopic data were measured using a Varian 660-IR spectrophotometer. NMR spectra were recorded using a Bruker NMR spectrometer (Bruker, Leipzig, Germany) with an operating frequency of 400 and 100 MHz, respectively. CDCl_3_ (Chloroform-d, Sigma Aldrich, St. Louis, MO, USA) was used as a solvent for all extracts. The chemical shifts were given in δ (ppm) and coupling constants (J) were reported in Hz. The chemical shifts obtained were compared with some of the isolated compounds from S. officinalis leaves (refer to Supplementary Table S1) [28]. All the spectra are shown in the Supplementary Material (Figures S1–S4).

### 2.4. Thin-Layer Chromatography (TLC) Analysis 

TLC was performed following the method of Gwatidzo et al. [31] with minor modifications. Five 5 × 5 cm TLC plates (Silica gel 60 F254, Supelco, Bellefonte, PA, USA) were used. Pencil lines were drawn 0.5 cm from one edge of each plate. Extract samples at 50 mg/mL were spotted (1 µL) using micropipettes onto the pencil lines. For comparison, three standards were used, including quercetin (Essential Nutrition, Monterrey, Mexico), 4-methylumbelliferone, and anthrone (Sigma Aldrich, St. Louis, MO, USA), each one at a concentration of 1 mg/mL. The plates were then placed in a development chamber with a trial solvent (chloroform (CCl_3_) or a solution of CCl_3_/methanol (MeOH). The plates were removed and the solvent front was marked with a soft pencil. The plates were visualized under UV light at 254 and 365 nm. As revealing reagents, the plates were sprayed with ceric sulfate, 1% ferric chloride, or a fine spray of 1% ethanolic aluminum chloride solution, respectively. The resultant chromatograms were captured on camera and the retention factor was calculated (Rf = distance traveled by the spot/distance traveled by the solvent front) (Figure S5). All plates were repeated at least in triplicate.

### 2.5. Total Flavonoid Content (TFC) in S. officinalis Leaf Extract 

The method described by Chang et al. in 2020 [32], with modifications from Wakeel et al. in 2019 [33], was employed. Dilutions of S. officinalis ethanolic extract (5 mg/mL methanol) were prepared, resulting in a final volume of 40 µL. Then, 20 µL of 10% AlCl_3_, 20 µL of CH_3_CO_2_K 1M, and 380 µL of H_2_O_2_ were added. The reaction was incubated for 30 min and then read at a wavelength of 405 nm using a spectrophotometer (BioMate, Thermo Fisher Scientific, Waltham, MA, USA). A standard curve was constructed with quercetin at concentrations of 50, 25, 12.5, and 6.25 µg/mL. The total flavonoids amount was expressed as the quercetin equivalent in µg/mg of extract.

### 2.6. Total Antioxidant (TAC) and Ferric-Reducing (FRPA) Capacity of S. officinalis Leaf Extract

The phosphomolybdenum assay [34,35] was employed. In brief, 20 µL of the *S. officinalis* ethanolic extract (5 mg/mL methanol) sample was mixed with 180 µL of a phosphomolybdenum stock (0.6 M sulfuric acid, NaH_2_PO_4_ 28 nM, 4 mM ammonium molybdate) and incubated at 95 °C for 90 min in a water bath. The mixture was then read at a wavelength of 630 nm using a spectrophotometer (BioMate, Thermo Fisher Scientific, Waltham, MA, USA). Ascorbic acid served as a positive control. The antioxidant capacity was calculated as follows: % antioxidant capacity = [1 − (OD of the sample/OD of the control)] × 100.

The potassium ferrocyanide–ferric chloride method [36,37], with some modifications, [33] was utilized to analyze the reducing capacity. Briefly put, 40 µL of the *S. officinalis* ethanolic extract (5 mg/mL methanol) sample was mixed with 50 µL of phosphate buffer (0.2 M, pH 6.6) and 50 µL of 1% potassium ferrocyanide, followed by incubation at 50 °C for 20 min. Subsequently, 50 µL of 10% trichloroacetic acid was added, and the mixture was centrifuged at 3000 rpm for 10 min. The top layer was collected, and 33.3 µL of 0.1% ferric chloride was added. The samples were then read at a wavelength of 630 nm using a spectrophotometer (BioMate Thermo). The reducing power was calculated using the formula: % reducing power = [1 − (OD of the sample/OD of the control)] × 100.

### 2.7. Determination of Total Phenolic Contents (TPC)

The total phenolic content was assessed using the Folin–Ciocalteu (FC) reagent assay, following the methodology of Jafri et al. (2017) [34], with some adaptations for microplate analysis based on Wakeel et al. (2019) [33]. A stock solution of FC reagent was created by diluting it from 2N to 1N with deionized distilled water. The assay involved adding 20 µL of the test sample and 90 µL of the FC reagent to each well of a 96-well plate. After incubating the plate for 5 min, 90 µL of a 20% sodium bicarbonate solution was added to each well. The plate was then incubated at room temperature for 90 min. Gallic acid, at final concentrations of 1000, 500, 200, 100, 40, 20, 10, and 5 µg/mL, served as the standard for the calibration curve (y = 0.4925x − 0.4479, r^2^ = 0.9937). Absorbance readings were taken at 760 nm using a microplate reader [38,39,40].

### 2.8. Preparation of Treatment Solutions of S. officinalis Leaf Extract

Ten milligrams of the ethanolic extract of S. officinalis was dissolved in 1 mL of dimethyl sulfoxide (DMSO) with shaking at room temperature. A 1 mg/mL dilution was prepared in Dulbecco’s Modified Eagle’s Medium-High Glucose (DMEM-HG, Biowest, Riverside, MO, USA) supplemented with 1× penicillin/streptomycin (Antibiotic–Antimycotic 100 x, Gibco, Thermo Fisher Scientific, Waltham, MA, USA). The solution was filtered with a 0.22 µm membrane (General Lab, TPP, Trasadingen, Switzerland), and concentrations of 75, 100, 125, 150, and 200 µg/mL were prepared in DMEM-HG supplemented with 1% (*v*/*v*) calf serum (Biowest, Riverside, MO, USA) and 1x penicillin/streptomycin. As a positive damage control, cisplatin (ACOCIT) was used at a concentration of IC_50_ of 58.3 µM (17.4 mg/mL) for 48 h [41]. DMEM-HG medium with 0.1% DMSO was used as a negative damage control, and DMEM-HG medium supplemented with 1% calf serum served as a blank control. 

### 2.9. Cell Culture

The TC-1 cell line (ATCC: CRL-2493; Manassas, VA, USA) was maintained in DMEM-HG supplemented with 10% (*v*/*v*) calf serum and 1x penicillin/streptomycin. Cells were incubated at 37 °C in 95% air in a humidified incubator with 5% CO_2_. In total, 0.25% Trypsin–EDTA Solution (Gibco, Thermo Fisher Scientific, Waltham, MA, USA) was used for cell harvesting during subculturing, following the conditions reported by Keshavarz et al. in 2020 [42]. This cell line has been extensively used as a model for studying cervical cancer due to its expression of HPV-16 E6 and E7 oncogenes, which are crucial in the pathogenesis of this disease [43]. The selection of the TC-1 model is grounded in its capability to mimic the immune response [44,45] and genetic [46] characteristics of HPV-16+ cervical cancer [46].

### 2.10. Viability Assay 

Cells were seeded in tissue culture (TC)-treated 96-well flat-bottom microplates (Corning^®^, Corning, NY, USA) at a density of 1 × 10^4^ cells/well in supplemented DMEM-HG, and incubated for 24 h. Afterward, the medium was removed and replaced with the treatments previously described in a final volume of 100 µL/well, and the cells were incubated for 48 h in a 5% CO_2_ atmosphere at 37 °C. At the end, the treatments were removed and replaced with 100 µL/well 1x Alamar Blue™ Cell Viability Assay Reagent (Sigma–Aldrich, St. Louis, MO, USA) in a phenol red-free medium for 4 h. The optical density was measured using a microplate reader (iMark, Bio-Rad, Hercules, CA, USA) at a wavelength of 570–600 nm for excitation emission. The percentage of cytotoxicity was calculated as = 100 − [(experimental OD value − blank OD value)/(control OD value − blank OD value) × 100%]. Images were captured with the AE31 EPI inverted microscope (Motic, Xiamen, China) coupled to the Moticam Pro 252A camera and Motic Images Plus 2 software.

### 2.11. Clonogenic Assay

Cells were plated in TC-treated 24-well flat-bottom microplates (Corning^®^, Corning, NY, USA) at a density of 500 cells/well in triplicate and incubated for 24 h. The medium was removed and replaced with a final volume of 500 µL of the treatments, and the cells were incubated for 48 h. Afterward, the treatment medium was aspirated and substituted with DMEM-HG medium supplemented with 10% calf serum, and the plates were incubated for 14 days with medium changes every 5 days. At the end of the incubation period, cells were fixed for 15 min with buffered 10% formalin, stained with 0.4% crystal violet for 45 min, washed with H_2_O to remove excess dye, and images were captured. For quantification, plates were incubated with 10% acetic acid for 1 h at room temperature with constant shaking. Optical density was measured using a microplate reader (iMark, Hercules, CA, USA) at a wavelength of 570 nm. Percentage survival was calculated as: (treatments OD × 100)/control OD.

### 2.12. Wound Healing Assay

Cells were seeded in TC-treated 24-well flat-bottom microplates (Corning^®^) at a density of 5 × 10^5^ cells/well and incubated for 24 h in a 5% CO_2_ atmosphere at 37 °C to reach approximately 90% confluence. Monolayers were scraped using a 200 μL tip, washed with 1x sterile PBS at pH 7.4, and incubated with serum-free treatments. Images were captured at 24 h and 48 h intervals using the AE31 EPI inverted microscope (Motic), equipped with the Moticam Pro 252A camera and Motic Images Plus 2 software, to monitor cell migration in the wounded area. Image analysis was performed using T Scratch software v1.0 [47], and the percentage area reduction was calculated using the formula: 100 − [(area Tx × 100)/area T0], where Tx = the area at 24 h or 48 h, and T0 = the area at baseline.

### 2.13. 3D-Spheroid Culture Formation and Reversion Assay

To generate the 3D-spheroid cultures, the liquid overlay technique was used [20,48]. Briefly put, in a 96-well plate, a non-adherent surface was created with 1% agarose, and 5000 cells were seeded in 100 µL of DMEM-HG medium supplemented with 10% calf serum. The cells were incubated for 96 h in an atmosphere containing 5% CO_2_ at 37 °C. Subsequently, the maintenance medium was replaced with treatments, and the 3D cultures were incubated for 48 h. To assess reversion to 2D cultures, spheroids were transferred to a TC-treated 96-well plate and incubated for 24 h in a 5% CO_2_ atmosphere at 37 °C. Then, the medium was replaced with maintenance DMEM-HG and incubated for 7 days. Images were captured at the beginning and end of treatments and at the end of reversion with the AE31 EPI inverted microscope (Motic, Xiamen, China), equipped with the Moticam Pro 252A camera and Motic Images Plus 2 software. The spheroid area measurement was performed using ImageJ software v1.38e, and changes in % growth were calculated using the formula: 100 − [(area T48 h × 100)/area T0].

### 2.14. 3D-Spheroid Culture Inhibition Assay

In a 96-well plate with a non-adherent 1% agarose surface, 5000 cells were seeded in 100 µL of the corresponding treatment media and incubated for 96 h. In the end, the spheroids were transferred to a TC-treated 96-well plate and incubated for 24 h in a 5% CO_2_ atmosphere at 37 °C, then the medium was replaced with maintenance DMEM-HG and incubated for 7 days. Images were captured at 96 h and 7 days, and the spheroid area was determined as previously described.

### 2.15. Statistical Analysis

The results are presented as mean ± standard deviations (SD). In the cytotoxic assay, 3D-spheroid formation, and inhibition of 3D-spheroid formation, the data represent the mean of three independent experiments with 10 replicates per experiment. For the clonogenic assay and the wound healing assays, results represent the mean of three independent experiments with three replicates per experiment. Group differences were evaluated using the Kruskal–Wallis statistical test with a Mann–Whitney U post hoc analysis. All statistical analyses were conducted using IBM SPSS Statistics 20 software. Statistical significance is denoted by the corresponding symbols in the figures for *p*-values < 0.0001, <0.001, <0.01, and <0.05.

## 3. Results

### 3.1. Ethanolic Extract of S.officinalis Leaves Has Flavonoids, Tannins, Steroids, and Saponins, and Has Reducing and Antioxidant Capacity

The preliminary phytochemical analysis revealed the presence of several secondary metabolites, such as tannins, flavonoids, steroids, and saponins in the ethanolic extract obtained from leaves. Likewise, the Dragendorff test was negative for alkaloids, while the Mayer and Wagner tests yielded positive results. This may be due to the fact that alkaloid types like pyrrolizidine, isoquinoline, and indole could react more strongly with the Mayer and Wagner tests (Table 1).

The UV analysis showed the presence at a maximum of 230, 281, and 330 nm (Figure S1). Regarding to the IR spectrum, signals were observed at 3400 cm^−1^ (O-H stretching), 1650 cm^−1^ (C=O), 1600 cm^−1^ (C=C), and 1200 to 1000 cm^−1^ (C-O-C) (Figure S2), which could correlate with signals for flavonoids [49] similar to catechin and quercetin, consistent with the UV spectrum where characteristic absorption peaks for this type of structure were observed. In order to try to identify some of these compounds, an NMR ^1^H and ^13^C NMR scanning analysis was conducted (Supplementary Figures S3 and S4). 

The comparison of the chemical shifts in the *S. officinalis* extract with nuclear magnetic resonance profiles of metabolites previously isolated from the leaves reveals some similarities (Supplementary Table S1). Some characteristic signals should be noted. 

Firstly, the peaks observed in the ethanol extract at δ ^1^H of 2.460, 2.48, 1.44, 0.974, and 0.779 ppm suggest the presence of aliphatic compounds or highly hydrocarbonated chains. These could belong to some fatty acids, terpenes, or metabolite skeletons such as carnosol, carnosic acid, rosmanol, and β-sitosterol, respectively. These have been previously reported for the Salvia family [50,51,52]. 

Secondly, in the ^1^H NMR spectrum, a set of signals was observed that appears as singlets at low fields (around 6–12 ppm). These signals could belong to protons in the flavanone-type metabolite skeletons similar to quercetin (Supplementary Table S1), metabolites previously isolated from Salvia species [53,54]. It is important to mention that, given the complexity of the ethanol extract, obtaining a clear profile using ^13^C NMR is difficult. However, in the spectrum obtained, signals around 110 to 120 ppm were observed, which could indicate carbons belonging to double bonds that might be associated with some of the aforementioned metabolites. 

To further clarify the presence of flavonoid-type metabolites, thin-layer chromatography (TLC) experiments were conducted using quercetin as a standard of flavonoid compounds, as well as other metabolites with similar chromophores like anthrone and 4-methylumbelliferone (Figure S5) [31,55]. The TLC profile of the SO ethanolic extract showed seven spots at the polarity 80:20, CCl_3_/MeOH. It is important to denote that the spots at Rf from 0.6 to 1 showed a positive reaction to the aluminum chloride and ferric chloride, reagents indicative of flavonoids and phenols, respectively. Also, these spots gave abortions under the UV light, showing a wine-red coloration (Figure S5C–E) [31]. Although the Rf value of the flavonoid standard (0.5) used did not match any spot in the extract at the evaluated polarity, we did not rule out the presence of a derivative. The presence of a derivative compound of quercetin or catechin were considered considering the signals observed in the NMR experiments, the UV and IR signals, along with the positive reaction to flavonoid reagents. Furthermore, these compounds could be glycosides, modifying the retention factor. Therefore, we propose that the evaluated extract is rich in flavonoid-type compounds, but further experiments using more sophisticated techniques (HPLC, GC-MS) are required to isolate and identify these metabolites [56,57,58]. 

Table 2 presents the results of various assays conducted on extracts obtained from the leaves of *S. officinalis*, specifically focusing on total flavonoid content (TFC), ferric-reducing power assay (FRPA), total antioxidant capacity assay (TAC) and total polyphenol content (TPC). These results indicate the presence of flavonoids in the extract, as evidenced by the quantified total flavonoid content and polyphenols. Additionally, the extract demonstrates significant ferric-reducing power and antioxidant capacity compared to the control (ascorbic acid). 

### 3.2. Ethanolic Extract of S. officinalis Affects the Viability and Replication of TC-1 Cells 

Initial tests were performed using concentrations of 1, 15, 25, 50, and 100 µg/mL. The experimental design was therefore refined using 75, 100, 125, 150, and 200 µg/mL concentrations. The upper limit of 200 µg/mL was set due to the appearance of a slight precipitate.

**Figure 1:**
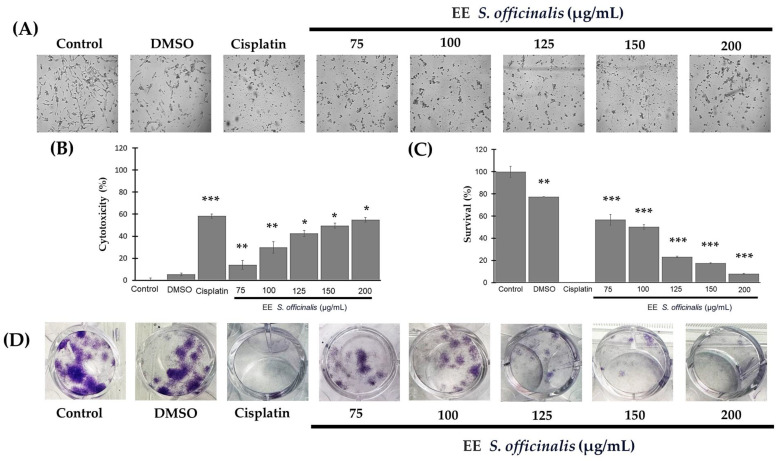
Ethanolic extract of *S. officinalis* leaves affects cell viability and clonogenic survival of TC-1 cells in a dose-dependent manner. (**A**) Microphographs show the morphological changes observed in TC-1 cells after treatment with ethanolic extract of *S. officinalis*. Magnification 10×. (**B**) The graph shows the concentration-dependent effect of *Salvia* ethanolic extract on cell viability at 48 h. (**C**) The graph shows the percentage of survival in each treatment. (**D**) Photographs show a decrease in colony-forming capacity compared to controls, highlighting the impact of *S. officinalis*. *p*-values corresp ond to significant differences compared to the control, DMEM-HG medium, alone, * *p* < 0.05, ** *p* < 0.01, *** *p* < 0.001.

At these concentrations, the ethanolic extract of *S. officinalis* leaves induced changes in the cell morphology of TC-1 cells within 48 h of exposure (Figure 1A). Starting from the lowest concentration of 75 µg/mL, a reduction in cell extensions was observed. The cells transitioned from fibroblast-like to rounded cells. At this concentration, there was also a significant difference in cell viability compared to the control, with a cytotoxicity percentage of 14 ± 0.3% (*p* < 0.05). Moreover, at higher concentrations of 100, 125, 150, and 200 µg/mL, this difference was maintained, resulting in values of cytotoxicity of 29.76 ± 5.23%, 42.44 ± 6.27%, 49.57 ± 2.19%, and 54.22 ± 1.35%, respectively (*p* < 0.001) (Figure 1B). Notably, the concentration of 200 µg/mL exhibited the highest inhibitory effect without a significant difference compared to cisplatin treatment, indicating a similar cytotoxic effect to the chemotherapeutic agent (*p* = 0.095).

The capacity of cells to give rise to another cell and form colonies was also affected by treatment in a concentration-dependent manner. Cisplatin treatment completely inhibited colony formation compared to the control, while treatment with 0.1% DMSO resulted in a survival of 77.42 ± 0.19% (*p* = 0.046). Treatment with *S. officinalis* showed a significant difference concerning the blank control from the lowest concentration (75 µg/mL), where 56.73 ± 4.84% (*p* < 0.010) survival was observed. This difference remained consistent at concentrations of 100, 125, 150, and 200 µg/mL, with survival percentages of 50.41 ± 2.08%, 23.18 ± 0.71%, 17.58 ± 0.33%, and 8.05 ± 0.36%, respectively (*p* < 0.001) (Figure 1C). Figure 1D illustrates the growth of cell colonies (purple color) in comparison to the treatment.

### 3.3. Ethanolic Extract of S. officinalis Leaves Inhibits TC-1 Cell Migration

**Figure 2:**
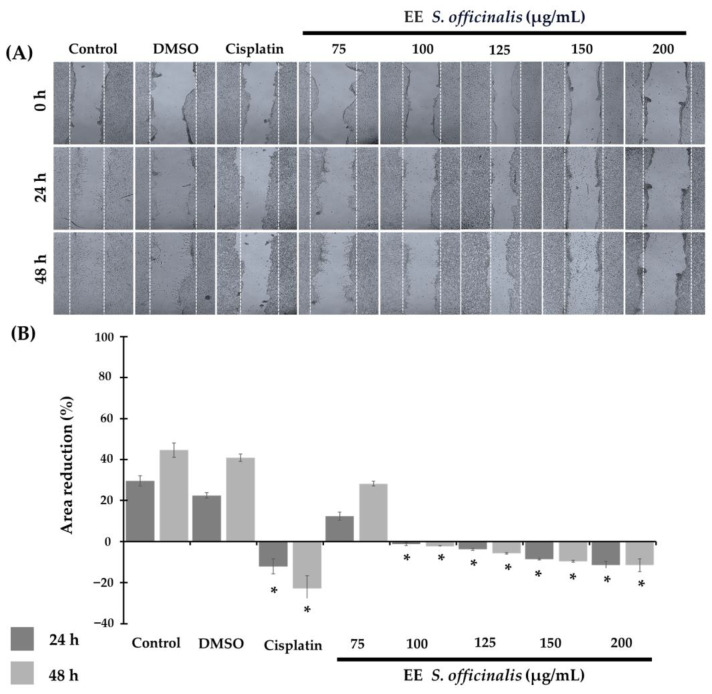
The migration capacity of TC-1 cells is inhibited by the ethanolic extract of *S. officinalis* leaves. (**A**) Microphotographs show the change in the wound opening area in TC-1 cell monolayers at 24 h and 48 h follow-up times. Magnification 4×. (**B**) The graph represents the percentage reduction in the wound area. It is observed that starting from the concentration of 100 µL/mL, the ethanolic extract of *S. officinalis* leaves inhibits migration compared to the blank control and DMSO control. * *p* < 0.05.

The impact of the ethanolic extract of *S. officinalis* leaves on TC-1 cell migration was evaluated at 24 h and 48 h (Figure 2A). At 24 h, the effect on migration was concentration-dependent. The blank control group showed a percentage reduction in the wound area of 29.5 ± 2.56%, while the DMSO control showed a reduction of 22.41 ± 1.30%, with no statistical difference between them (*p* = 0.07). The extract at 75 µg/mL also induced a reduction of 12.34 ± 1.90% but was statistically different from the control and cisplatin (*p* = 0.05). Instead, from 100 µg/mL, an increase in wound size was observed: 1.3 ± 0.64% at 100 µg/mL, 3.88 ± 0.38% at 125 µg/mL, 8.68 ± 1.61% at 150 µg/mL, and 11.5 ± 1.70% at 200 µg/mL. In other words, a significant reduction in cell migration was observed starting at the 100 µg/mL concentration compared to the blank control group and the DMSO control (*p* = 0.05) (Figure 2B).

At 48 h, the effect persisted in all experimental groups. In the blank control group and the DMSO control, the wound area reduction increased by 48.85 ± 1.70% and 44.61 ± 3.50%, respectively, with no statistical difference between them (*p* = 0.127). Also, the treatment with 75 µg/mL maintained the decrease in the wound area, although in a lower percentage than the controls, with 20.85% ± 1.18 (*p* = 0.05) concerning the blank control and DMSO control. At the higher concentrations of 100, 125, 150, and 200 µg/mL, the increasing trend in the wound area was maintained: 2.32 ± 0.36%, 5.77 ± 0.39%, 9.67 ± 0.39%, and 11.57 ± 3.50%, respectively (*p* = 0.05), concerning the blank control and DMSO control. This was similar to the results observed in cisplatin-treated cells that at 24 h showed a significant increase in wound size of 12.0 ± 3.6% and persisted at 48 h with a 22.9 ± 6.24% rise. Interestingly, no difference was found between the treatment with cisplatin and the ethanolic extract of *S. officinalis* at 200 µg/mL at 24 h (*p* > 0.812) and 48 h (*p* < 1.12).

### 3.4. Ethanolic Extract of S. officinalis Decreases the Growth of 3D Cultures of TC-1 and Their Reversion to 2D Cultures

**Figure 3:**
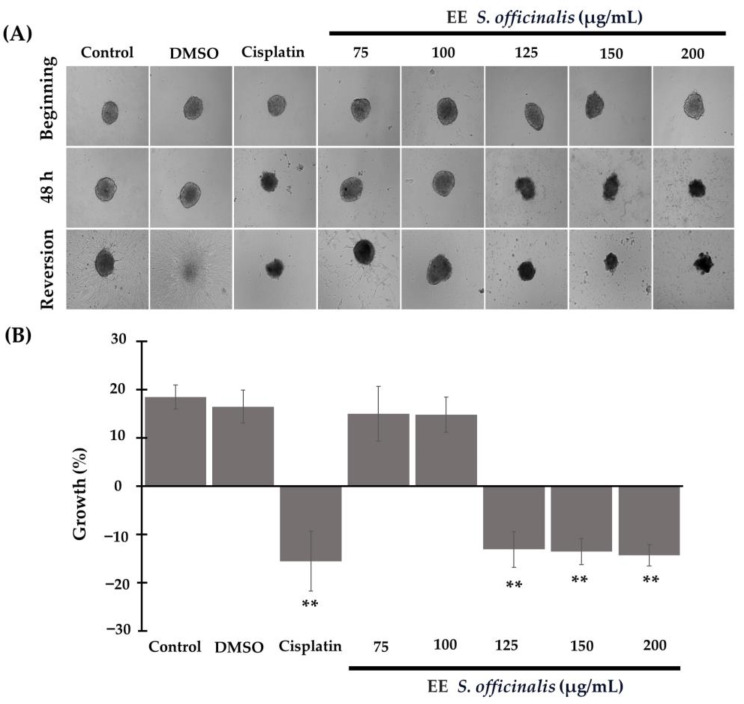
The ethanolic extract of *S. officinalis* leaves inhibits the growth and reversion of 3D cultures of TC-1 cells in a dose-dependent manner. (**A**) The microphotograph in the upper panel shows the initial state of the spheroids before treatment. Magnification 10×. The central panels show changes after 48 h of incubation with the different treatments. In the lower panel, micrographs illustrate the reversion of 3D cultures to 2D. (**B**) The graph represents the percentage change in the size of the spheroids after treatment, ** *p* < 0.01.

In 3D cultures of TC-1 cells, it was determined that exposure to the ethanolic extract of *S. officinalis* led to a significant reduction in their growth (Figure 3A). Compared to the DMSO control and blank control, from the concentration of 125 µg/mL, these were reduced by 13.08 ± 3.68% from 13.53 ± 2.47% at 150 µg/mL, and 14.33 ± 2.22% at the concentrations of 200 µg/mL (*p* < 0.001). When compared to cisplatin, which had a reduction of 15.5 ± 3.40%, there was no significant difference between cisplatin and the extract-treated groups (*p* = 0.126, *p* = 0.361, *p* = 0.221, with respect to 125, 150, and 200 µg/mL) (Figure 3B). In other words, the concentrations of 125, 150, and 200 µg/mL had a similar effect to the chemotherapeutic agent.

In the reversion assay, it was evident that treatment with the ethanolic extract of *S. officinalis* inhibited the adherence capacity of the cells at a concentration of 100 µg/mL and above, similar to cisplatin (Figure 3A). In contrast, spheroids treated with the blank control, DMSO control, and S. officinalis at 75 µg/mL adhered to the surface and proliferated, although the latter to a lesser extent (Figure 3A).

### 3.5. Ethanolic Extract of S. officinalis Inhibits the Formation of 3D Cultures of TC-1 and Their Reversion to 2D Cultures

**Figure 4:**
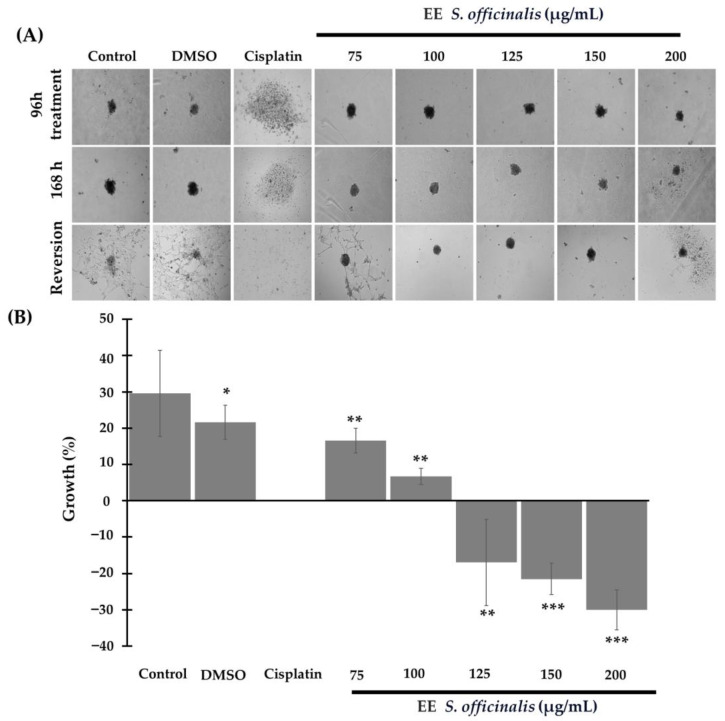
The pre-treatment of 3D cultures of TC-1 cells with the ethanolic extract of *S. officinalis* leaves inhibits their formation and reversion. (**A**) The microphotograph in the upper panel shows the development of 3D cultures under different treatments. Magnification 20×. The central panels show growth changes after removing the treatments and maintaining in the culture medium for 7 days, while the lower panel corresponds to the reversion and growth of 2D cultures. (**B**) The graph represents the percentage of 3D-spheroids growth (* *p* <0.05, ** *p* <0.010, *** *p* < 0.001).

The results revealed that pre-treatment with the blank control, DMSO control, and ethanolic extract of *S. officinalis* at 75 µg/mL did not affect spheroid growth (Figure 4). The growth percentages were 29.64 ± 11.85% and 21.61 ± 4.74% for the blank control and DMSO control, respectively, while for *S. officinalis*, it was 16.55 ± 3.44%. These groups had no statistical difference from the blank control, *p* = 0.386 and *p* = 0.088, respectively (Figure 4B).

In contrast, 3D cultures pre-treated with *S. officinalis* extract showed a significantly lower percentage growth from the concentrations of 100, 125, and 150 µg/mL compared to the blank control and DMSO control (*p* < 0.01) (Figure 4). At a concentration of 200 µg/mL, the percentage of spheroid growth was reduced by 21.80 ± 8.30% (*p* < 0.001), while cisplatin treatment had a 100% inhibitory effect in spheroids formation. Additionally, it was observed that pre-treatment with *S. officinalis* extract prevented cells from reverting to monolayer growth from the concentration of 100 µg/mL, unlike cells treated with the blank control and DMSO control (Figure 4A).

## 4. Discussion

In this study, it was determined that the ethanolic extract of *Salvia officinalis* leaves obtained using 96% ethanol exhibited effects on some of the hallmarks of cancer in the tumorigenic HPV-16+-related cancer cell line. This extract induced concentration-dependent changes in morphology, shortened cell prolongations, decreased viability, reduced clonogenic survival/replication, and inhibited migration. Likewise, when spheroids in 3D cultures were treated with the extract, it decreased their growth and ability to revert to 2D, while as a pre-treatment it also, in a concentration-dependent manner, inhibited the formation of 3D cultures. 

In this study, we employed the TC-1 cell line. This was derived from primary lung cells of C57BL/6 mice that were immortalized with HPV-16 E6 and E7 genes and then transformed with the pVEJB-expressing activated human c-Ha-ras gene [59]. This cell line is tumorigenic and is commonly used to induce tumor formation in mice [60,61]. That is why a validated model for testing the efficacy of therapeutics against HPV-associated neoplasia has been employed as a study model for cervical cancer [61,62,63]. This model allows the immune response to be examined in the context of the whole organism, closely mimicking the human response to cancer [45]. It is therefore used in preclinical studies of potential treatments such as vaccines against this cancer [61,62,63]. Consequently, our choice of this cellular model and this in vitro characterization correspond to the first phase to subsequently carry out studies using the in vivo mouse model. The aim is to assess the overall therapeutic effect of the ethanolic extract of sage leaves, its regulation of the pro-inflammatory response established in the cancer and tumor environment, as well as to establish its safety.

To the best of our knowledge, there are no previous assays that had evaluated the effect of this type of extract on tumorigenic HPV-related cancer cells. However, studies have been conducted where it was determined that both the essential oil and hydroalcoholic extracts (80% EtOH) from the leaves have antiproliferative and cytostatic effects on HeLa cells, superior to those shown by the aqueous extract [19,23]. In this context, our results are consistent with what has been observed in this line of human cervical cancer cells, although they differ in concentrations and exposure time. With the TC-1 cell line, decreased viability at 48 h of 50% was observed at 200 µg/mL of the extract, while Garcia et al. reported a similar effect at 24 h with 75 µg/mL of their hydroalcoholic extract and Privitera et al. at 48 h with 100 µg/mL [19,23]. While the differences may be due to both cell line characteristics and differences in extraction methods, it is clear that *S. officinalis* has cytotoxic effects on these types of cervical cancer lines. Our results also suggest interference with cell division, limiting the unlimited division capacity of these cells. Tannins, flavonoids, saponins, and steroids in the extract used in this study could be the effectors of the inhibitory activity on cell viability and clonogenic survival. It has been described that flavonoids modulate the activity of ROS-scavenging enzymes, arrest the cell cycle, induce apoptosis, and inhibit the proliferation of cancer cells [21,64]. The probable presence of epicatechin, carnosol, carnosic acid, rosmanol, and β-sitosterol suggested by nuclear magnetic resonance analysis in the ethanolic extract of *S. officinalis* leaves aligns with previous reports [65]. Some of these constituents have already been associated with specific biological activities; for example, carnosol and carnosic acid are associated with antioxidant activity [65,66].

The potential effects of the ethanolic extract of *Salvia officinalis* leaves on cell replication and migration can be hypothesized based on preliminary phytochemical studies and a general spectroscopic profile. Our studies have revealed that the extract is rich in flavonoid compounds, probably rich in previously identified substances, such as quercetin and catechin, as well as luteolin derivatives, which are some of the primary metabolites present in the Salvia family [67]. For instance, luteolin has been documented to exert notable antiproliferative and anti-migration effects. This compound achieves these through multiple mechanisms, including interference with cell cycle progression, induction of apoptosis, and inhibition of migration by modulating crucial signaling pathways, such as PI3K/Akt and NF-κB [68,69]. The effectiveness of luteolin is further enhanced by its structural features, particularly the presence of multiple hydroxyl groups, which contribute to its strong antioxidative and biocidal properties [69,70,71].

Similarly, it should be noted that studies, both in HeLa cells as well as in other cellular models of different types of cancer, report morphological changes consistent with what was observed in our study [19,23,72]. The authors report rounding and the formation of apoptotic bodies. Although we observed cell rounding, we consider that this could be related to changes in the cytoskeleton of the cell, since from the lowest concentrations (with less effect on viability), the morphological changes are seen. This hypothesis also aligns with the results of the migration assay, where the inhibition of this cancer hallmark was pronounced as early as 24 h and since the concentration of 100 ug/mL. This effect on migration was also previously observed by Keshavarz et al. in human umbilical vein endothelial cells at a 200 µg/mL concentration. These authors concluded that this is a specific effect on cell migration not related to viability or apoptosis [73]. Although we did not analyze cell viability during migration, the monolayers looked undamaged after 24 h, and the concentrations tested were lower than those reported by Keshavarz et al., so it could be inferred that viability would not be affected at 24 h; however, more research needs to be conducted to clarify this point [71]. Therefore, we postulate that the ethanolic extract of S. officinalis could have a direct effect on the cytoskeleton and/or signaling pathways regulating cell migration. For example, methanolic extracts of S. rosmarinus leaves have been found to inhibit the migration of HGUE-C-1, HT-29, and SW480 cells [74]. Furthermore, it has been reported that luteolin and quercetin flavonoids, previously identified in the S. officinalis extracts, can regulate this cellular process [67,69]. In this regard, quercetin has been shown to impact it by downregulating the expression of matrix metalloproteinases (MMPs) such as MMP9 and MMP2, which play a significant role in the degradation of the extracellular matrix, thereby inhibiting the migration and invasive potential of cancer cells [71]. Therefore, the effect of Salvia ethanolic extract on invasion, another hallmark of cancer, needs to be characterized, as there are no previous studies on this subject. Taken together, these data suggest that Salvia species contain phytochemicals with inhibitory activity in this cellular process. However, further research is essential to characterize and isolate these compounds; for example, using tools to analyze the entire pool of molecules present in complex mixtures, such as metabolomics and the mass spectral networking for natural products [10]. In this way, we can fully understand their mechanisms, potentially leading to novel therapeutic approaches for treating diseases characterized by excessive cell proliferation and migration. In our approach to exploring the impact of *S. officinalis* on tumors in vitro, we evaluated its activity in 3D cultures of these cells. In the formed spheroids, we observed that the extract was concentration-dependent when inhibiting tumor growth. Remarkably, when applied as a pre-treatment, the extract limited cell aggregation and spheroid formation. Importantly, no reversion to a 2D format was observed even at the lowest concentration tested (75 µg/mL). This suggests that the extract induced either cell death throughout the aggregate or the inhibition of factors related to cell adhesion. These data highlight the potential therapeutic utility of *S. officinalis* extract in modulating cell aggregation and tumor growth, as well as its potential role in combination strategies with conventional chemotherapeutic agents.

In this regard, corroborative evidence from animal studies further supports our findings. For instance, in a murine tumor model of Ehrlich ascites carcinoma, a hydroalcoholic extract of *Salvia officinalis* was used as an adjuvant with doxorubicin, contributing to a decrease in tumor volume with fewer toxic effects in mice [75]. These findings not only bolster the therapeutic potential observed in our in vitro studies, but also highlight the importance of conducting further research to confirm whether these results can be effectively replicated in in vivo systems. Additionally, the cell line employed in our study can serve as a foundation for establishing a robust animal model, which would allow for a more comprehensive evaluation of the therapeutic potential and safety profile of *Salvia officinalis* extracts [61,73]. Considering these encouraging findings, future studies are planned to further characterize the effect of *Salvia officinalis* on this type of tumor using in vivo models.

All the results of this work allow us to postulate that the ethanolic extract of *Salvia officinalis* leaves, particularly at a concentration of 200 µg/mL, exerts an antitumor effect; further investigations are imperative to validate this hypothesis. Detailed analyses of the extract’s metabolites, exploration of signaling pathways and/or genes it might be modulating, and assessments of its impact on other cancer hallmarks, such as invasion and angiogenesis, should be pursued. Likewise, it should be considered that, although the extract demonstrated inhibitory effects, its activity did not surpass that of the cisplatin used as a control. Therefore, the evaluation of higher concentrations or additional analyses should be considered to determine whether the extract could be used as an adjuvant to reduce the dosage of chemotherapy agents that typically carry severe adverse effects.

Regarding the possible mechanism of action of the ethanolic extract of *Salvia officinalis* leaves on the HPV virus, it was previously reported that the diterpenoid naphthoquinone called tanshinone IIA, present in formulations of *Salvia* spp. commonly used in traditional Chinese medicine, decreases the expression of oncogenes E6 and E7 in Caski and SiHa cells (with HPV-16 integrated into their genome) [76,77]. In Caski cells, it was also characterized as causing S-phase cell cycle arrest and p53-mediated apoptosis [76], while in SiHa cells, it induces apoptosis by decreasing levels of the anti-apoptotic protein Bcl-2 and inhibits glycolysis by regulating the hypoxia response factor HIF-1α through the Akt/mTOR pathway [77]. This suggests that there may be compounds in the ethanol extract of *Salvia officinalis* leaves that can regulate the expression of HPV oncogenes and thereby prevent the malignant process or induce the death of already malignant cells. Further studies are therefore needed to establish the precise mechanisms.

Finally, this work has some limitations. Firstly, it is important to mention that while the use of this specific cell line has provided valuable insights, particularly in understanding the interactions between *S. officinalis* extracts and HPV-16+-related cervical cancer processes, a limitation of our study is the reliance on a single cell type for our assays. Generally, research incorporating multiple types of cell lines, including non-tumorigenic counterparts, can provide a more comprehensive assessment of a compound’s efficacy and safety, enhancing the reliability and applicability of the results. Another limitation is that transwell assays were not performed in our experimental setup, which would have allowed us to analyze possible changes in the invasive behavior of TC-1 cells. It is important to note that in the present study, we did not perform analysis using HPLC or GC-MS, which would be important to consider in future experiments due to the significance of identifying the unequivocal structure of the metabolites present in the extract, particularly those with Rf values of 0.6 to 0.9 (Figure S5), as they exhibited significant UV absorptions in the TLC analysis. Furthermore, comparative studies with other parts of the plant, such as roots and flowers, which may have similar effects, should be considered. In-depth studies are also necessary when transitioning to preclinical in vivo assays to track marker metabolites of the species.

Despite these limitations, our study has made significant contributions to the field, demonstrating that the ethanolic extract of *Salvia officinalis* leaves can induce concentration-dependent changes in cell morphology, reduce viability, inhibit clonogenic survival, and prevent migration and spheroid formation in HPV-16+ cancer cells. These findings are particularly noteworthy, as they suggest multiple mechanisms of action, from affecting cell structure and viability to impacting cellular aggregation and migration, which are crucial for cancer progression. This discovery paves the way for future research to delve deeper into the selectivity of the extract and its potential applications in cancer treatment.

## 5. Conclusions

The ethanolic extract of *S. officinalis* leaves with a content of tannins, flavonoids, steroids, and saponins, and a reducing capacity of 45% and antioxidant capacity of 44%, reduced the viability of tumorigenic HPV-16+-related cancer cells in a concentration-dependent manner. In addition, it decreased the replication capacity and particularly inhibited the migration and growth of 3D cultures and their reversion to 2D. As a pre-treatment, the extract interfered with the formation of 3D cultures and completely inhibited their reversion. This suggests that the phytochemicals present in the extract have biological activity against HPV-related cancers, so more exhaustive and specific analyses should be carried out to establish which are the active compounds and the mechanisms of action that modulate these hallmarks of cancer.

## Figures and Tables

**Table 1:** Preliminary phytochemical analysis of the ethanolic extract *of Salvia officinalis* leaves.

Metabolites	*S. officinalis* Ethanolic Extract *
Tannins (FeCl_3_)	++
Tannins (gelatin hydrolysis)	++
Flavonoids (Shinoda test)	++
Flavonoids (Salkowski test)	+++
Steroids	++
Alkaloids (Dragendorff test)	-
Alkaloids (Wagner test)	+
Alkaloids (Mayer test)	+
Saponins (hemolysis in agar)	+++
Saponins (foam formation)	+
Coumarins (NaOH test)	-

+++ = appreciable amount (positive within 5 min); ++ = moderate amount (positive after 5 min but within 10 min); + = trace amount (positive after 10 min but within 15 min); - = completely absent. * Concentration of extract at 5 mg/mL.

**Table 2:** Total flavonoid content, ferric-reducing capacity assay, total antioxidant capacity, and total polyphenol content of extracts of *S officinalis* leaves.

	*S. officinalis* Ethanolic Extract *
TFC	QE = 153.4074074 ± 10.68 µg/mg extract
FRPA	% age reduction = 45.62 ± 5.30
TAC	% age TAC = 44.57 ± 1.27
TPC	GAE = 89.08 ± 3.01 µg/mg extract

TFC: expressed in quercetin equivalents (QE) µg/mg of extract. FRPA: expressed as % reducing power relative to the ascorbic acid control. TAC: expressed as % antioxidant capacity relative to the ascorbic acid control. TPC: expressed in µg of gallic acid (GAE) for mg of extract. * Concentration at 5 mg/mL.

## Data Availability

The datasets used and/or analyzed during the current study are available from the corresponding author upon reasonable request.

## Supplementary Materials

The following supporting information can be downloaded at: https://www.mdpi.com/article/10.3390/biomedicines12081804/s1, Figure S1. UV spectra recorded in MeOH at 0.1 mg/mL. Figure S2. Infrared spectra (wavenumber in cm^−1^). Figure S3. 1 H-NMR spectra (400 MHz) of leaves of Salvia officinalis, recorded in CDCl3. Figure S4. 13C NMR spectra (100 MHz) of leaves of Salvia officinalis, recorded in CDCl3. Figure S5. Thin-layer chromatography (TLC) analysis of the ethanol extract of Salvia officinalis and reference compounds. The figure displays five TLC plates: (A) chromatography under short-wave UV light (254 nm), (B) chromatography under long-wave UV light (365 nm), (C) visualization with 1% ethanolic aluminum chloride solution at 365 nm, (D) visualization with ceric sulphate, and (E) visualization with 10% ferric chloride (FeCl_3_, 1.0%). The samples analyzed include EE: ethanol extract of Salvia officinalis (50 mg/mL), Q: quercetin (1 mg/mL), A: anthrone (1 mg/mL), and 4-ML: 4-methylumbelliferone (1 mg/mL). The solvent system used was 8:2 chloroform/methanol. Table S1. Comparisons of chemical shifts of the SO extract vs. some of the principal metabolites isolated from the leaves of Salvia species.
