# Supplementary material for: Ethanolic Extract of Salvia officinalis Leaves Affects Viability, Survival, Migration, and the Formation and Growth of 3D Cultures of the Tumourigenic Murine HPV-16+-Related Cancer Cell Line
Source: Biomedicines. 2024 Aug 8;12(8):1804. doi: 10.3390/biomedicines12081804 (PMC11351782; doi:10.3390/biomedicines12081804)
Supplement: Supplementary file 1 [file biomedicines-12-01804-s001.zip › biomedicines-3121616-supplementary.pdf]

Suplementary files

Ethanolic Extract of *Salvia officinalis* Leaves Affects Viability, Survival, Migration, and the Formation and Growth of 3D Cultures of the Tumourigenic Murine HPV-16+-Related Cancer Cell Line

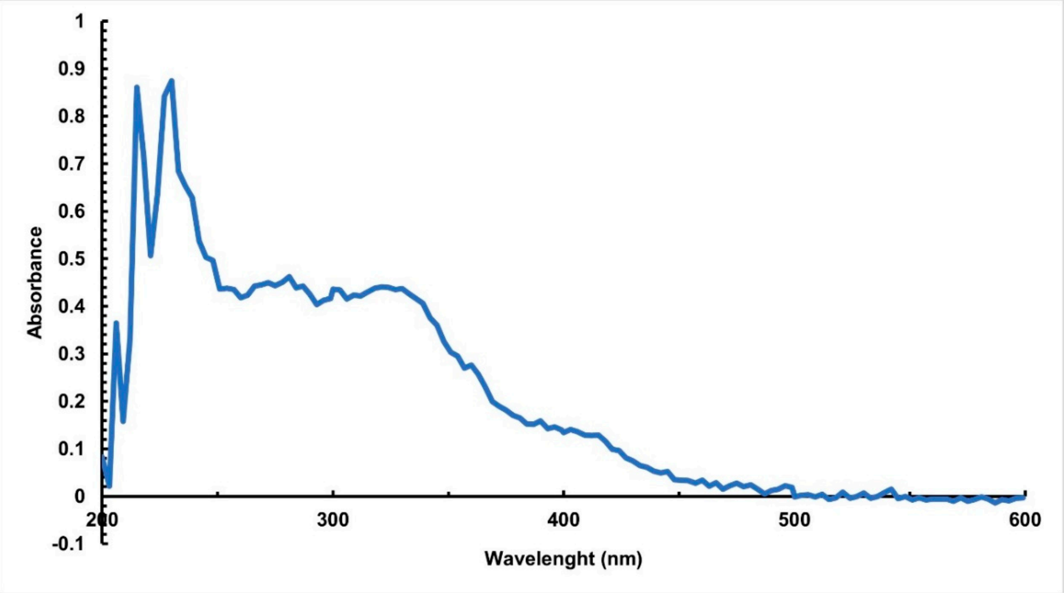

Figure S1. UV spectra recorded in MeOH at 0.1mg/mL.

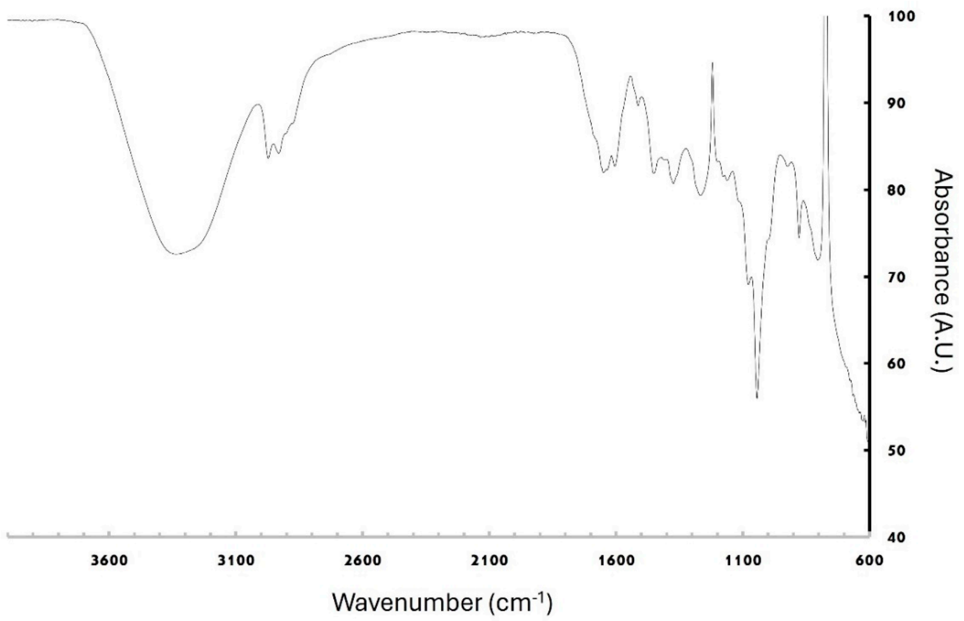

Figure S2. Infrared spectra (wavenumber in cm^{-1}).

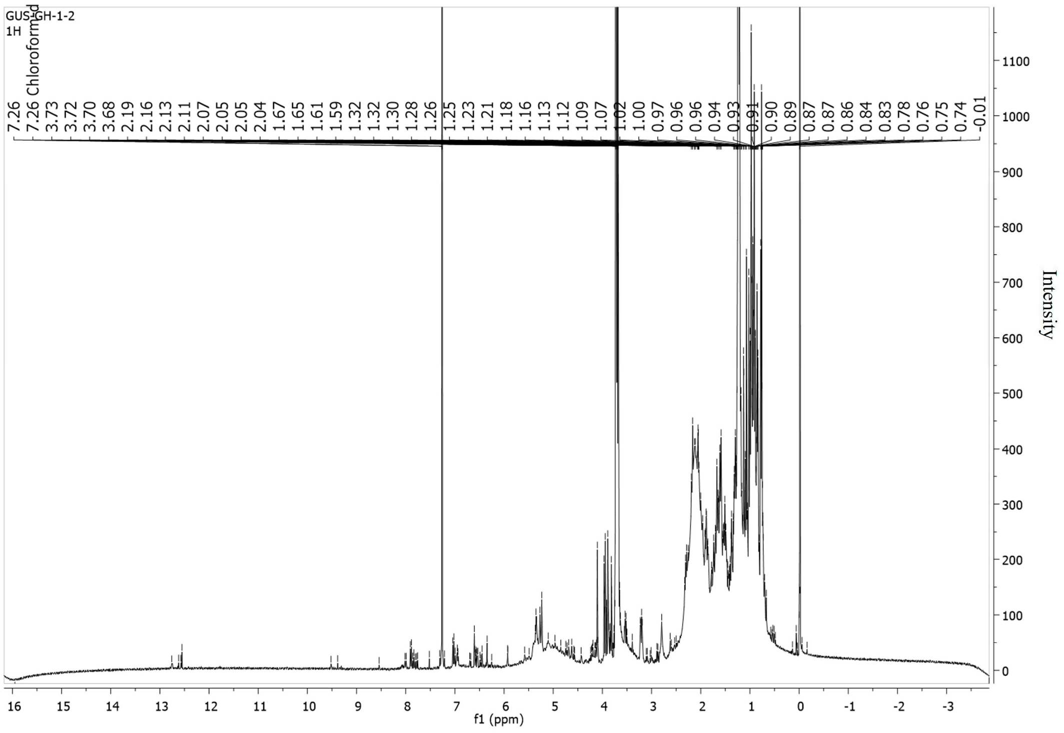

Figure S3. ^{1}H-NMR spectra (400 MHz) of leaves of *Salvia officinalis*, recorded in $CDCl_3$.

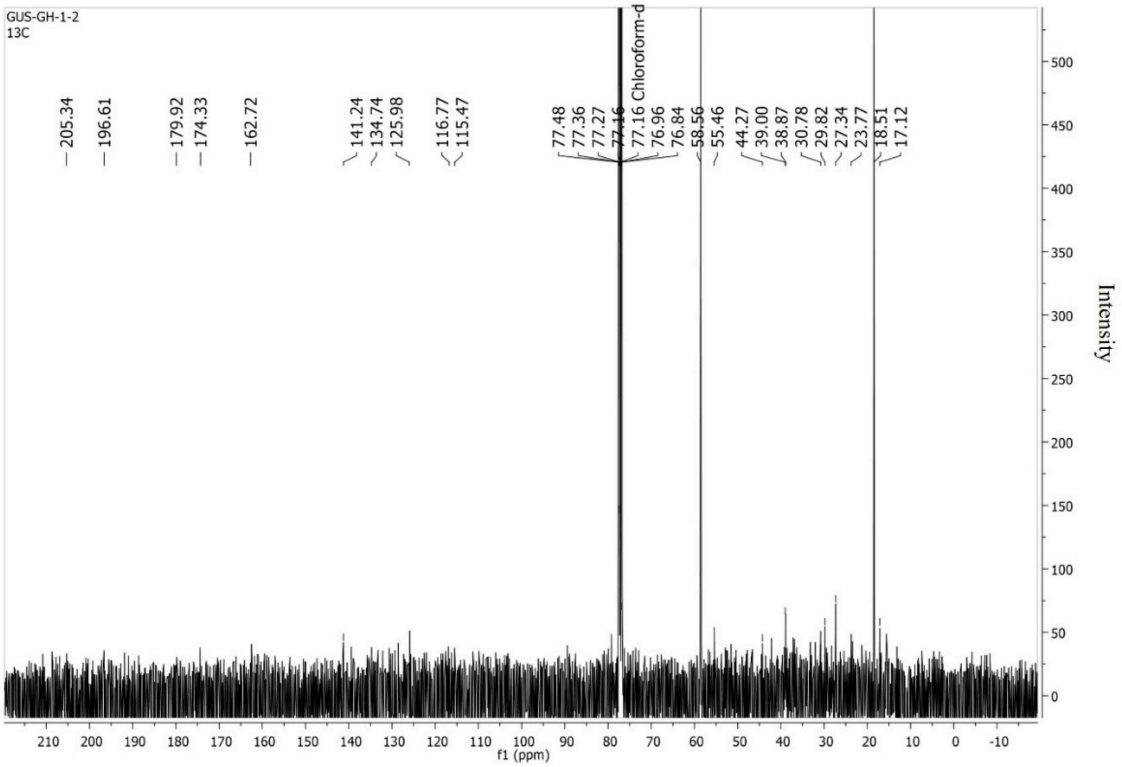

Figure S4. ^{13}C NMR spectra (100 MHz) of leaves of *Salvia officinalis*, recorded in $CDCl_3$

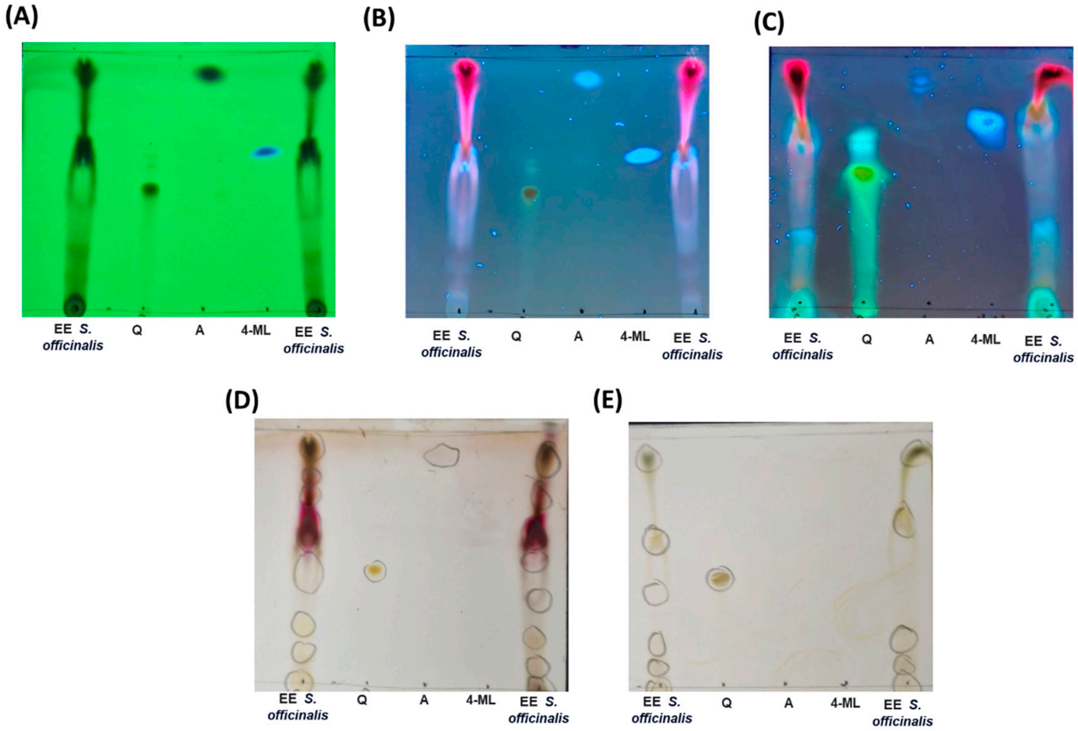

Figure S5. Thin-layer chromatography (TLC) analysis of the ethanol extract of Salvia officinalis and reference compounds. The figure displays five TLC plates: (A) chromatography under short-waved UV light (254 nm), (B) chromatography under long-waved UV light (365 nm), (C) visualization with 1% ethanolic aluminum chloride solution at 365 nm, (D) visualization with ceric sulphate, and (E) visualization with 10% ferric chloride ($FeCl_3$, 1.0%). The samples analyzed include EE: ethanol extract of Salvia officinalis (50 mg/mL), Q: quercetin (1 mg/mL), A: anthrone (1 mg/mL), and 4-ML: 4-methylumbelliferone (1 mg/mL). The solvent system used was 8:2 chloroform/methanol.

Table S1. Comparisons of chemical shifts of the SO extract vs some of the principal metabolites isolated from the leaves of Salvia species

Signal	SO extract*		Catechin*		Quercetin**		Carnosol*		Carnosic Acid*		Rosmanol*		β-sitosterol*	
δ	^{1}H	^{13}C	^{1}H	^{13}C	^{1}H	^{13}C	^{1}H	^{13}C	^{1}H	^{13}C	^{1}H	^{13}C	^{1}H	^{13}C
1	0.764 (s)	15.58	4.56 (d)	82.68	6.22 (s)	147.4	2.36 (ddd) 2.71 (ddd)	28.6	1.17 3.26 (br.d)	34.4	1.94 (ddd) 3.11 (br.d)	27.3	0.86-1.10 (m)	11.9
2	0.779 (s)	15.73	4.06 (m)	68.30	6.44 (s)	136.2	1.51 (dddd) 1.81 (dddd)	18.6	1.73 (br.d) 1.56 (m)	20.3	1.46 (m) 1.39 (m)	19.1	3.53 m	12.1
3	0.912 (d)	18.51	2.53 (dd)	28.80	7.70(s)	176.4	1.39 (ddd) 1.13 (ddd)	40.7	1.29 (ddd) 1.44 br. (d)	41.6	1.11 (br.d) 1.37 (m)	38.2	5.35 (m)	18.9
4	0.974 (s)	21.30	2.98 (dd)	100.60	6.91(d)	161.2	1.6 (dd)	34.2	1.53 (br.d)	34.5	2.15 (s)	31.3		19.1
5	1.21 (s)	23.77	6.02 (d)	157.17	7.58 (d)	98.7	2.06 (ddd) 1.75 (dddd)	45.3	2.37 (m) 1.82 (m)	53.9	4.49 (d)	50.8		19.5
6	1.23 (s)	27.34	5.88 (dd)	96.10	9.18 (s)	164.4	5.26 (ddd)	29.5	2.79 (m)	18.9	4.66 (d)	78.2		19.9
7	1.24 (s)	29.81	6.90 (d)	157.70	12.46 (s)	93.8	6.51 (s)	78.1	2.79 (m)	31.5	6.08 (s)	68.5		21.2
8	1.58 (br.s)	30.78	6.8 (d)	95.38	10.70 (s)	157.0	3.07 (sept)	131.6	6.53 (s)	129.0	3.07 (sept)	128.1		23.1
9	2.79 (m)	38.67	6.76 (dd)	156.65	9.18 (s)	98.7	1.06 (d)	121.5	3.14 (sept)	122.6	1.16 (d)	124.5		24.4
10	3.18-3.23 (dd)	39.00		132.13	9.18 (s)	122.5	1.05 (d)	48.2	1.17 (d)	48.7	1.13 (s)	47.2		26.2
11	3.69 (q)	44.27		115.19		115.6	0.75 (s)	142.3	1.17 (d)	142.3	0.95 (s)	142.9		28.3
12	3.81 (s)	55.46		145.60		145.5	0.72 (s)	142.4	0.97 (s)	141.2		141.8		29.2
13	3.89 (s)	58.56		147.70		116.1		134.2	0.87 (s)	133.6		135.1		31.7
14	3.94 (s)	76.96		115.64		115.7		111.6		119.1		120.3		31.9
15	3.96 (s)	77.27		119.99		148.2		26.6		27.0		27.3		32
16	4.10 (s)	77.36						22.4		22.5		22.1		34
17	4.62 (s)	115.47						22.4		22.1		22.6		36.2

18	5.23 (t)	125.98						31.4		32.6		31.4		36.6
19	5.27 (t)	134.74						19.3		21.35		22.3		37.3
20	5.6.35 (s)	141.24						177.5		182.1		179.8		39.8
21	5.93 (d)	162.72												42.3
22	6.35 (s)	174.33												42.4, 46
23	6.44 (s)	179.92												50.2
24	6.60 (s)	196.61												56.1
25	6.94 (m)	205.34												56.8
26	7.02 (s)													71.9
27	7.04 (s)													121.8
28	7.52 (s)													140.8
29	7.76 (s)													
30	7.78 (s)													
31	7.81 (s)													
32	7.83 (s)													
33	7.86 (s)													
34	7.88 (s)													
35	7.91 (s)													
36	8.54 (s)													
37	9.38 (s)													
38	9.57 (s)													
40	12.55 (s)													
41	12.57 (s)													
42	12.62 (s)													

43	12.76 (s)													

[a]δ from TMS (ppm). ^{1}H NMR [400 MHz, $CDCl_3$, J (Hz)] and ^{13}C NMR (100 MHz) data for SO extract. br (broad signal). Chemical shifts obtained from x (40,41), Y (40,41) and Z (42). *$CDCl_3$ as a solvent. ** DMSO-d 6 as a solvent
